# Mesenchymal stromal cells‐derived small extracellular vesicles modulate DC function to suppress Th2 responses via IL‐10 in patients with allergic rhinitis

**DOI:** 10.1002/eji.202149497

**Published:** 2022-04-24

**Authors:** Ya‐Qi Peng, Zi‐Cong Wu, Zhi‐Bin Xu, Shu‐Bin Fang, De‐Hua Chen, Hong‐Yu Zhang, Xiao‐Qing Liu, Bi‐Xin He, Dong Chen, Cezmi A. Akdis, Qing‐Ling Fu

**Affiliations:** ^1^ Otorhinolaryngology Hospital The First Affiliated Hospital Sun Yat‐sen University Guangzhou China; ^2^ Department of Otolaryngology‐Head and Neck Surgery Guangdong Provincial People's Hospital Guangdong Academy of Medical Sciences Guangzhou China; ^3^ Swiss Institute of Allergy and Asthma Research (SIAF) University of Zurich Davos Switzerland; ^4^ Christine Kühne – Center for Research and Education (CK‐CARE) Davos Switzerland; ^5^ Key Laboratory for Stem Cells and Tissue Engineering Ministry of Education Sun Yat‐Sen University Guangzhou China

**Keywords:** Small extracellular vesicles, Mesenchymal stromal cells, Dendritic cells, Allergic rhinitis, Interleukin‐10

## Abstract

Mesenchymal stromal cells (MSCs) are well known for their immunoregulatory roles on allergic inflammation particularly by acting on T cells, B cells, and dendritic cells (DCs). MSC‐derived small extracellular vesicles (MSC‐sEV) are increasingly considered as one of the main factors for the effects of MSCs on immune responses. However, the effects of MSC‐sEV on DCs in allergic diseases remain unclear.

MSC‐sEV were prepared from the induced pluripotent stem cells (iPSC)‐MSCs by anion‐exchange chromatography, and were characterized with the size, morphology, and specific markers. Human monocyte‐derived DCs were generated and cultured in the presence of MSC‐sEV to differentiate the so‐called sEV‐immature DCs (sEV‐iDCs) and sEV‐mature DCs (sEV‐mDCs), respectively. The phenotypes and the phagocytic ability of sEV‐iDCs were analyzed by flow cytometry. sEV‐mDCs were co‐cultured with isolated CD4^+^T cells or peripheral blood mononuclear cells (PBMCs) from patients with allergic rhinitis. The levels of Th1 and Th2 cytokines produced by T cells were examined by ELISA and intracellular flow staining. And the following mechanisms were further investigated.

We demonstrated that MSC‐sEV inhibited the differentiation of human monocytes to iDCs with downregulation of the expression of CD40, CD80, CD86, and HLA‐DR, but had no effects on mDCs with these markers. However, MSC‐sEV treatment enhanced the phagocytic ability of mDCs. More importantly, using anti‐IL‐10 monoclonal antibody or IL‐10Rα blocking antibody, we identified that sEV‐mDCs suppressed the Th2 immune response by reducing the production of IL‐4, IL‐9, and IL‐13 via IL‐10. Furthermore, sEV‐mDCs increased the level of Treg cells.

Our study identified that mDCs treated with MSC‐sEV inhibited the Th2 responses, providing novel evidence of the potential cell‐free therapy acting on DCs in allergic airway diseases.

## Graphical abstract

**MSC‐sEV regulated the differentiation and function of dendritic cells in allergic rhinitis**.  Peripheral monocytes treated with GM‐CSF, IL‐4, and LPS differentiated into dendritic cells. DCs treated with MSC‐sEV inhibited the type 2 immune response from allergic rhinits. sEV‐mDC exhibited a regulatory phenotype by the production of IL‐10 and induced Treg cells. GM‐CSF: granulocyte‐macrophage colony‐stimulating factor, LPS: lipopolysaccharide, mDCs: mature dendritic cells, MSC: mesenchymal stromal cells, sEV: small extracellular vesicles, Treg: regulatory T.

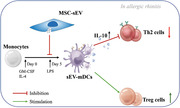

## Introduction

Mesenchymal stromal cells (MSCs) are known to be potent immunoregulators in immunological diseases [1], including allergic diseases [2, 3]. We previously reported that MSCs generated from induced pluripotent stem cells (iPSCs) have a higher proliferative capacity, lower cell senescence, and lower immunogenicity compared to bone marrow‐derived MSCs (BM‐MSCs) [4, 5, 6]. These data all suggested potential clinical applications for iPSC‐MSCs with more homogeneity, large population, and easy quality control in the future. Moreover, we reported that iPSC‐MSCs prevented both eosinophilic [7] and neutrophilic [8] allergic airway inflammation in mouse models, and exerted immunomodulation on helper T cells with mechanisms including working on regulatory T cells [9, 10], dendritic cells (DCs) [5], and epithelial cells [11] via cell‐cell contact, soluble factors, or mitochondrial transfer.

Currently, the small extracellular vesicles (sEV) derived from MSCs have been increasingly suggested as a possible mechanism contributing to the effects of MSCs on tissue remodeling and regeneration as well as immunomodulation [12, 13]. Therefore, MSC‐sEV acquired similar immunomodulatory properties to their parental cells but without potential side effects induced by cell transplantation. MSC‐sEV carry active proteins, lipids, mRNAs, long non‐coding RNAs, or microRNAs [14, 15], which allows them to exhibit the immunomodulatory effects on the immune cells such as T cells [16], B cells [17], and natural killer (NK) cells [18]. It has been observed that MSC‐derived sEV suppress the inflammatory responses and the expression of inflammatory cytokines in mice model of atopic dermatitis [19]. MSC exosomes were reported to promote proliferation and immune‐suppression capacity of Tregs on peripheral blood mononuclear cells (PBMCs) from patients with asthma [20]. Furthermore, we recently reported that sEV derived from iPSC‐MSCs, but not fibroblasts significantly inhibited group 2 innate lymphoid cell (ILC2) function in vitro using the blood of patients with allergic rhinitis, and prevented allergic airway inflammation in ILC2‐dominant mice model via miR‐146a‐5p [21].

DCs are one of the most important APCs, driving naïve T cells into T helper 2 cells in the pathogenesis of allergic diseases. We have previously shown that human iPSC‐MSCs inhibited DC differentiation, and allowed them to acquire a phenotype of regulatory DCs [5]. To date, only a few studies are focusing on the effects of MSC‐sEVs on DC functions. Reis et al. reported that EVs derived from human BM‐MSCs attenuated the maturation and function of monocyte‐derived DCs [22]. Nevertheless, we still do not know about the effects of sEV derived from iPSC‐MSCs on the Th2 immune response initialized by DCs in the pathogenesis of allergic rhinitis (AR).

In this study, we isolated sEV from iPSC‐MSCs using anion exchange chromatography and investigated their effects on DC function to T cells derived from patients with AR. We found that MSC‐sEV inhibited the differentiation of DCs and increased the phagocytic capacity of sEV‐mature DCs (sEV‐mDCs). Furthermore, sEV‐mDCs suppressed the production of Th2 cytokines from AR patients via IL‐10 in vitro. Moreover, sEV‐mDCs exhibited their immunomodulatory effects by promoting the expansion of Treg cells. Collectively, our study shed a light on the potential cell‐free therapy of MSC‐sEV to allergic rhinitis.

## Results

### Characterization of MSC‐derived sEV

**Figure 1:**
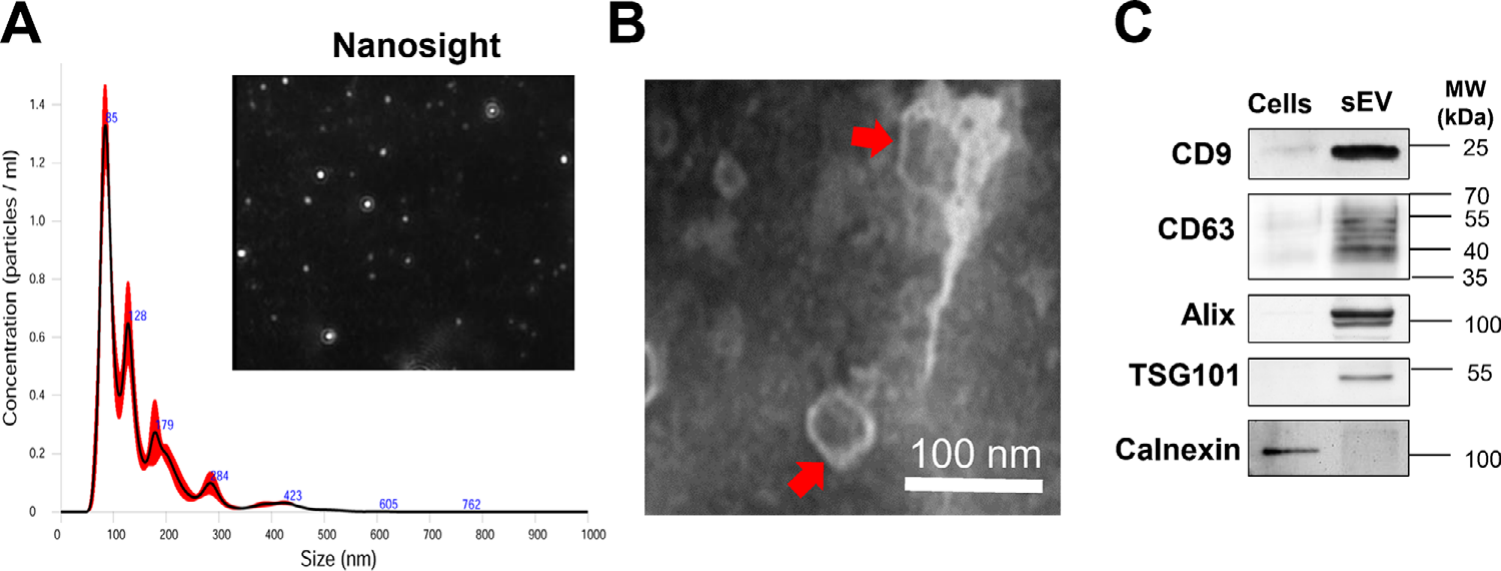
Characterization of iPSC‐MSC‐derived sEV. MSC‐sEV were isolated from conditioned medium by anion‐exchange chromatography. (A) Representative results of particle size distribution and nanoparticle tracking analyses of the sEV (1:100 dilution with particle‐free PBS). (B) The morphology of the nano‐size vesicles photographed by TEM. (C) sEV markers of CD9, CD63, Alix, and TSG101 were positive and Calnexin was negative in the sEV derived from MSCs as determined by western blot. All of the assessments have been repeated in at least three independent experiments. Right lane, MW markers. The lanes were cropped for presentation purposes, and the raw data were shown in the Supporting Information. iPSC: induced pluripotent stem cell, MSC: mesenchymal stromal cells, MW: molecular weight, sEV: small extracellular vesicles; TEM: transmission electron microscope. Scale bar, 100 nm.

In this study, iPSC‐MSC‐sEV were prepared and characterized as we previously reported [21]. MSC‐sEV purification yielded about 8 × 10^10^/mL and showed a mean size of approximately 100 nm by nanosight (Fig. 1A). sEV derived from iPSC‐MSCs were confirmed by transmission electron microscope (TEM) as diameters of 150 nm or less and exhibited the characteristic lipid‐bilayer (Fig. 1B). Then, we determined the definite expressions of EV markers CD9, CD63, Alix, and TSG101 using western blot. As expected, we found that the levels of all the markers were remarkably higher in MSC‐sEV compared to their parent cells. Importantly, the endoplasmic reticulum membrane marker, Calnexin, was found to be positive in the MSCs and negative in the MSC‐sEV (Fig. 1C and Supporting Information Fig. S1). Thus, we identified the characterization of sEV derived from iPSC‐MSC both in size and the expression of classic markers.

### MSC‐sEV inhibited the differentiation of CD14^+^ monocytes into DCs

**Figure 2:**
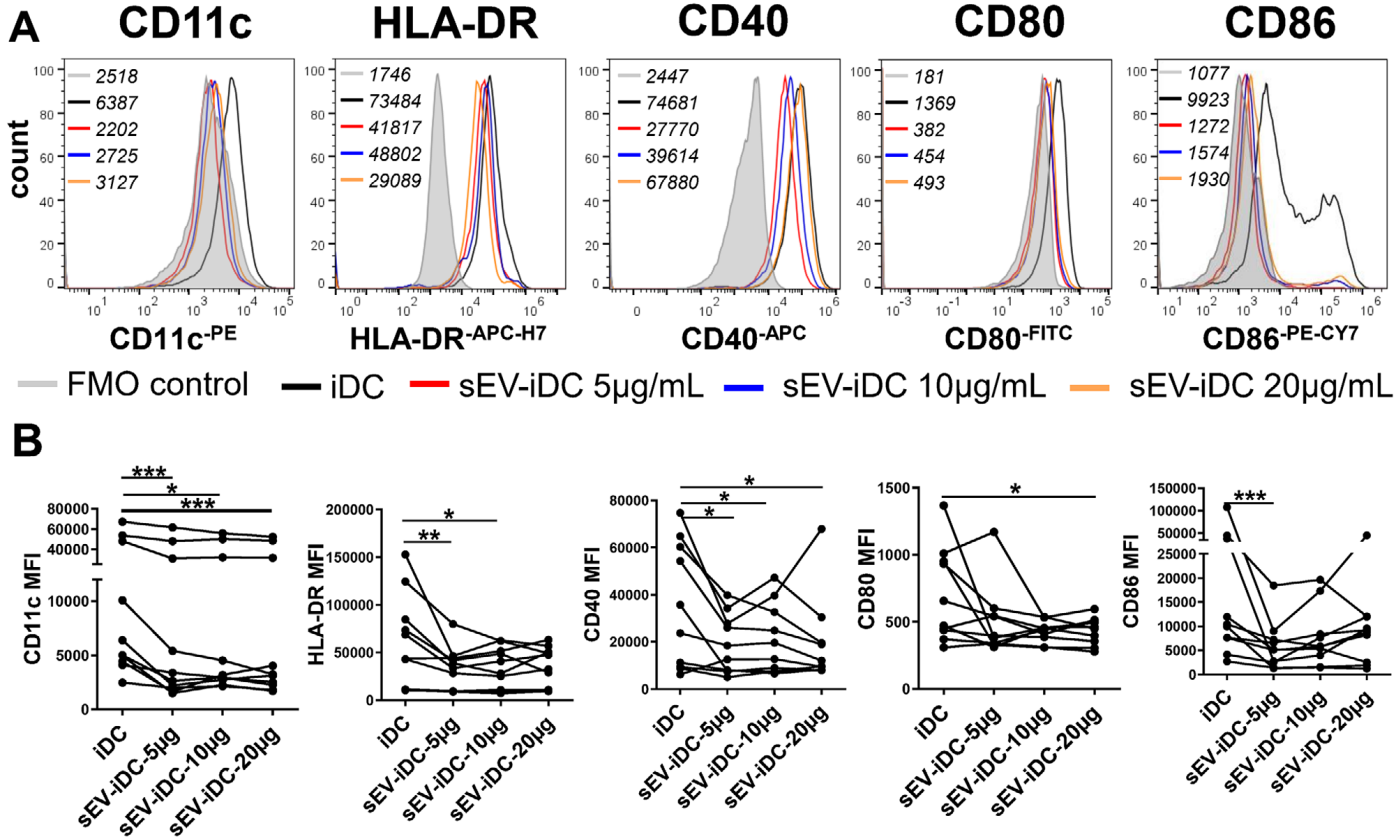
MSC‐sEV inhibited the differentiation of CD14^+^ monocytes into DCs. Sorted CD14^+^ monocytes were cultured in the presence or absence of MSC‐sEV (5, 10 or 20 μg/mL) for 5 days. (A) DC markers of CD11c, HLA‐DR, CD40, CD80, and CD86 were determined by flow cytometry. MFI values for different conditions were indicated top left in the figures. (B) Mean fluorescence intensity of the markers. The total amount of donors was *n* = 10, as human blood buffy coats from “anonymous donors.” Data are presented as mean ± SEM, and are from three independent experiments with three to four donors per experiment. Significance was determined comparing all sEV‐iDC samples to iDC without MSC‐sEV treatment. *p < 0.05, **p < 0.01, and ***p < 0.001. Statistical comparisons were performed using Tukey's multiple comparisons test. DCs: dendritic cells; FMO: fluorescence minus one, MSC: mesenchymal stromal cells, sEV: small extracellular vesicles.

To demonstrate the effects of MSC‐sEV on the differentiation of CD14^+^ monocytes, iDCs and sEV‐immature DCs (sEV‐iDCs) were generated as stated in the absence or presence of MSC‐sEV for 5 days. MSC‐sEV altered the phenotype of iDCs by reducing the expression of cell surface markers. The gating strategies are shown in Supporting Information Fig. S2. sEV‐iDCs expressed a significantly lower level of CD11c, HLA‐DR, CD40, CD80, and CD86 compared to their iDCs counterpart in different dosages (Fig. 2 and Supporting Information Fig. S3A). Representative pictures are also shown in Supporting Information Fig. S3A. These results were consistent with the potency of their parent cells as our previous report [5]. However, no differences were observed regarding the expression of CD14, CD1a, and CD141 (Supporting Information Fig. S3B). Both iDCs and sEV‐iDCs exhibited low expression of CD14, suggesting that MSC‐sEV suppressed the differentiation of iDCs rather than blocking DC differentiation from monocytes at the beginning.

### MSC‐sEV promote the mDC phagocytic ability but do not affect the expression of maturation markers

**Figure 3:**
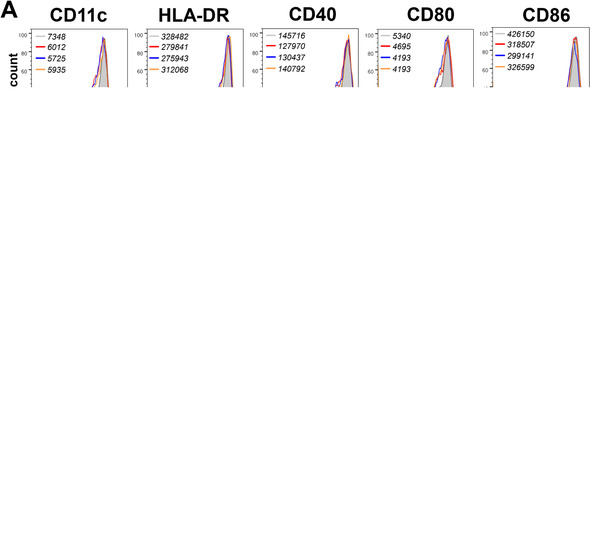
MSC‐sEV promoted the phagocytic ability of mDCs but did not affect the maturation surface markers. iDCs were treated with LPS in the presence or absence of MSC‐sEV for an additional 2 days. (A) DC markers of CD11c, HLA‐DR, CD40, CD80, and CD816 were determined by flow cytometry. MFI values for different conditions were indicated top left in the figures. (B) Bar graphs of the (MFI) of the markers. (C) Phagocytic ability of the iDCs, mDCs, and sEV‐mDCs was analyzed by flow cytometry, and the black line presents the expression of FITC‐Dextran at 4°. (D) Bar graph of the ΔMFI (37‐4°) of FITC‐dextran. Data are representative of collated data of four donors (A, B) or three donors (C, D) (mean + SEM). Statistical comparisons were performed using Tukey's multiple comparisons test. iDCs: immature dendritic cells, LPS: lipopolysaccharide, MFI: mean fluorescence intensity, mDCs: mature dendritic cells, MSC: mesenchymal stromal cells, sEV: small extracellular vesicles.

To further confirm the effects of MSC‐sEV on DC maturation, we induced the generation of sEV‐mDCs in vitro and determined the expression of DC surface markers. However, we did not find any differences in the phenotypic maturation between mDCs and sEV‐mDCs (Fig. 3A and B), which is consistent with our previous study about the effects of MSCs on DCs [5]. Here, sEV‐mDCs acquired a typical phenotype of mature DCs, by expressing CD11c, HLA‐DR, CD40, CD80, and CD86, regardless different concentrations of the MSC‐sEV added. These findings suggested that MSC‐sEV did not affect the expression of maturation markers from iDCs to mDCs.

The capacity of DCs to take up and process antigens is highly dependent on the stage of differentiation of DCs. Notably, iDCs have more strong ability to phagocytose dextran compared to mDCs. Therefore, we conducted the phagocytosis assay to study the influence of MSC‐sEV on the phagocytic ability of mDCs. As expected, iDC‐D7, the iDCs without the treatment of lipopolysaccharide (LPS) were harvested on day 7, was more powerful in the uptake of FITC‐dextran than mDCs. Furthermore, MSC‐sEV improved the ability of mDCs to take up FITC‐dextran but without significant difference (Fig. 3C and D, *p* = 0.0579). Generally, it indicated that sEV‐mDC acquired a capability of immune tolerance similar to iDCs but without any change in the phenotype.

Additionally, to test the lymphocyte‐stimulating ability of sEV‐mDCs, we conducted a mixed lymphocyte culture (MLC) system. sEV‐mDCs induced T‐cell proliferation in parallel to mDCs (Supporting Information Fig. S4), suggesting that MSC‐sEV had no effects on the ability of mDCs on T‐cell proliferation.

### MSC‐sEV impair the priming of mDCs on type 2 immune response from patients with AR in vitro

**Figure 4:**
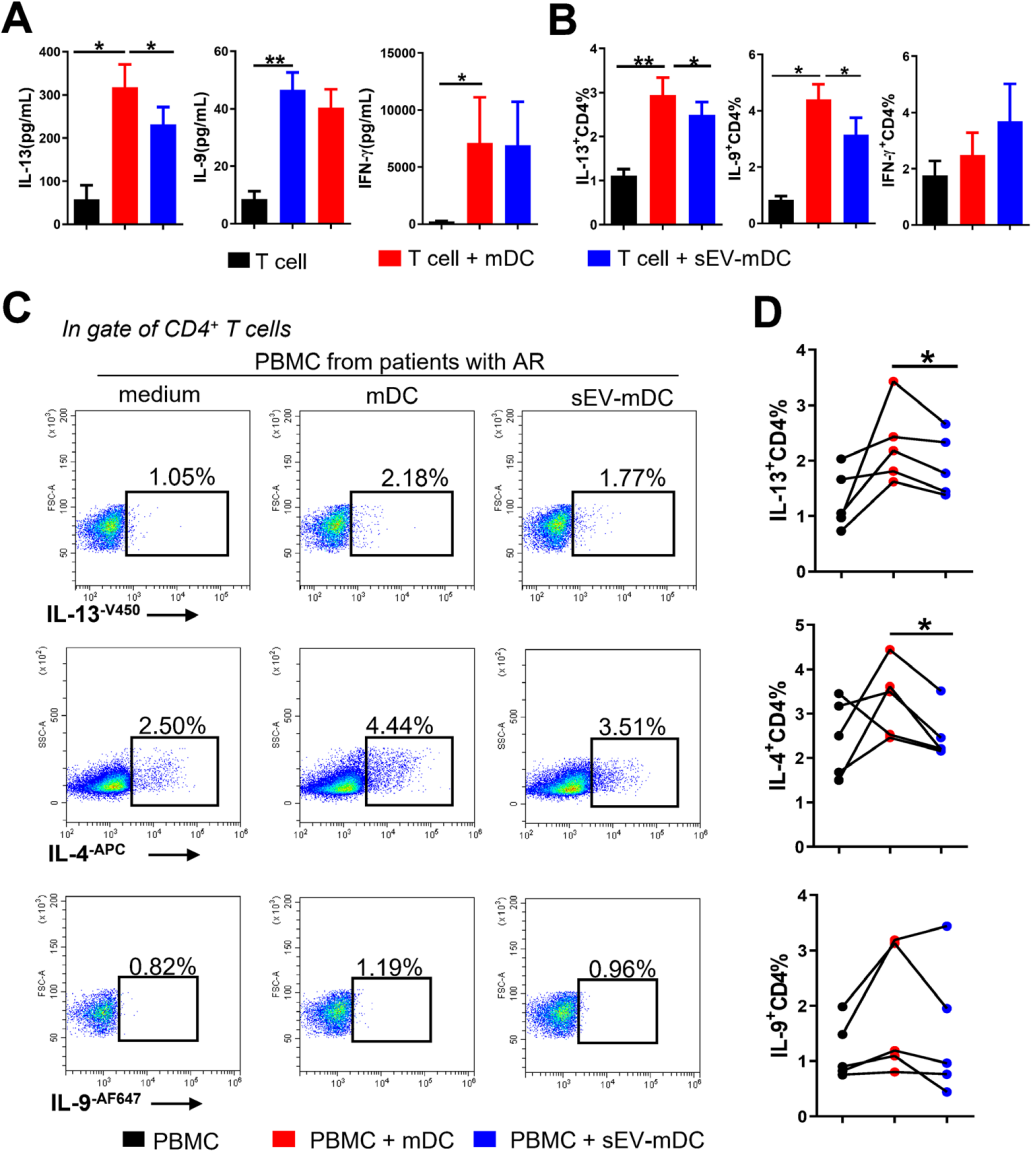
MSC‐sEV impaired the type 2 immunity priming capacity of mDCs in patients with AR in vitro. sEV‐mDCs were co‐cultured with sorted CD4^+^ T cells from buffy coat (A and B) or PBMCs from patients with AR (C and D) for 3 days. (A) The levels of IL‐13, IL‐9, and IFN‐γ in the supernatants of the co‐cultures. (B) The percentage of intracellular IL‐13, IL‐9, and IFN‐γ in T cells after co‐cultures with conditioned DCs (*n* = 6). (C and D) The percentages of IL‐13^+^ T cells, IL‐4^+^ T cells, and IL‐9^+^ T cells (*n* = 5). Data are representative of collated data of six donors for T cells isolation and three donors for DC generation, all from buffy coat (A, B), or five donors from patients with AR for PBMC and three donors for DC generation from buffy coat (C, D) (mean + SEM). *p < 0.05 and **p < 0.01, Tukey's multiple comparisons test. mDCs: mature dendritic cells, MSC: mesenchymal stromal cells, sEV: small extracellular vesicles.

To investigate whether MSC‐sEV affect the function of mDCs, human CD4^+^ T cells were isolated and co‐cultured with allogeneic sEV‐mDCs for 5 days. As we did not observe any dose‐dependent differences of MSC‐sEV on the maturational markers of DCs, we used the lowest dosage of MSC‐sEV with 5 μg/mL in the following functional experiments. We found that T cells produced lower IL‐13 but not IL‐9 and IFN‐γ when co‐cultured with sEV‐mDC compared with those co‐cultured with mDCs (Fig. 4A). We further observed a significant decrease in the percentage of IL‐13^+^CD4^+^ T cells and IL‐9^+^CD4^+^ T cells after co‐cultured with sEV‐mDCs compared to their mDC counterpart (Fig. 4B, Fig. S2B). Recently, IL‐9‐producing T cells were considered as a new T helper (Th) cell subset, instead of a subgroup of Th2 cells, namely Th9 cells [23]. In addition, IL‐9 was reported as the most enriched gene in allergen‐specific T cells from patients with asthma [24]. Taken together, these findings suggested that the treatment of MSC‐sEV to mDCs impaired their type 2 immunity enhancing ability, but did not affect the development of type 1 immune response in vitro. Moreover, MSC‐sEV might have the potential to suppress the function of Th9 cells in parallel.

mDCs were recognized to present the allergens to the naïve T cells and induce them into Th2 cells or Th9 cells in the pathogenesis of allergic diseases [25, 26]. To further examine the function of sEV‐mDCs, human PBMCs from patients with AR were co‐cultured with allogeneic sEV‐mDCs. Similar results were found that the levels of intracellular IL‐13 and IL‐4 in CD4^+^ T cells but not IL‐9^+^CD4^+^ T cells were decreased after the treatment of sEV‐mDCs (Fig. 4C and D). Overall, these findings suggest that MSC‐sEV could mitigate the function of mDCs in promoting Th2 cells from patients with AR in vitro.

### IL‐10 is responsible for the immunomodulatory effects of sEV‐mDCs

**Figure 5:**
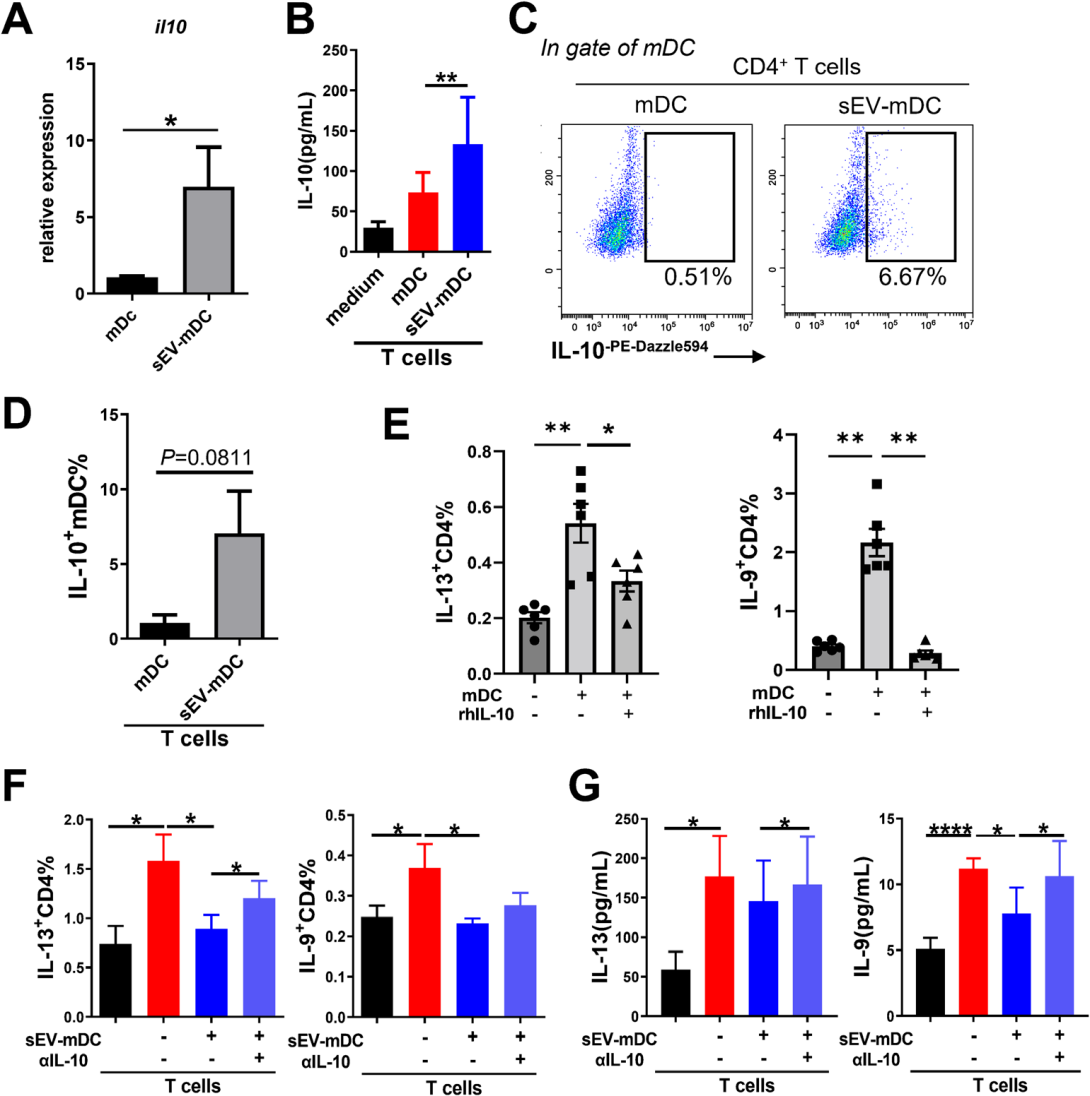
IL‐10 was responsible for the immunomodulatory effects of sEV‐mDCs. (A) The relative expression of IL‐10 mRNA in mDCs treated with or without MSC‐sEV. (B) The level of IL‐10 in the supernatants of DC‐T cell co‐cultures. (C and D) The level of intracellular IL‐10 in mDCs after co‐cultured with sorted CD4^+^ T cells from buffy coat. (E) The levels IL‐13^+^CD4^+^T cells and IL‐9^+^CD4^+^T cells after treated with rhIL‐10 (10 ng/mL). (F) The levels of intracellular IL‐9 and IL‐13 in T cells in the addition of anti‐IL‐10 antibody to the co‐cultures. (G) The levels of IL‐9 and IL‐13 in the supernatants of co‐cultures with the administration of anti‐IL‐10 antibody. Data are representative of collated data of nine donors (A), or eleven donors for T cell isolation and seven donors for DC generation (B–G), all from buffy coat (mean ± SEM). *p < 0.05, **p < 0.01, and ****p < 0.0001, Wilcoxon matched‐pairs signed‐rank test (A), paired *t*‐test (D) and Tukey's multiple comparisons test (B, E, F, and G). mDCs: mature dendritic cells; MSC: mesenchymal stromal cells; sEV: small extracellular vesicles.

DCs can exert their function on T cells by the secretion of a series of soluble inflammatory cytokines. Therefore, to address the potential mechanism underlying the immunomodulatory effects of sEV‐mDCs, we examined the mRNA levels of the main functional cytokines after the treatment of MSC‐sEV. We observed an increased mRNA level of IL‐10 in sEV‐mDC compared to mDCs (Fig. 5A). Meanwhile, no difference was observed in the mRNA levels of IL‐12p70, TNF‐α, IL‐1β, and OX‐40L (encoded by *tnfsf4*) (Supporting Information Fig. S5). The production of IL‐10 is a certain characteristic of the regulatory DCs. Then we first identified that there was higher level of IL‐10 in the supernatants of sEV‐mDCs co‐cultured with T cells compared to those of mDCs (Fig. 5B). To confirm the activity of DCs to produce IL‐10, we next examined the intracellular level of IL‐10 in DCs in the co‐culture system. We found that there was higher level of IL‐10^+^mDCs after T cells were co‐cultured with sEV‐mDCs but not mDCs, but without significant difference (*p* = 0.0811, Fig. 5C and D), which indicated that MSC‐sEV promoted mDCs to get the function of regulatory DCs, and induced the production of IL‐10 in sEV‐mDCs. We then checked the effects of rhIL‐10 on the activation of mDCs on CD4^+^ T cells. We found that rhIL‐10 significantly reversed the activate effects of mDCs on T cells as inhibiting the production of IL‐13 and IL‐9 (Fig. 5E and Supporting Information Fig. S6A). Taken together, these data suggest that MSC‐sEV treatment promoted the production of IL‐10 from mDCs, resulting in the suppression of type 2 immune response.

To further confirm the function of IL‐10 secreted by sEV‐mDCs in inhibiting Th2 immunity, we conducted the co‐culture experiments of T cells with conditioned DCs by the addition of the IL‐10‐neutralizing antibodies, and the levels of IL‐13 and IL‐9 were examined. As expected, the treatment of neutralizing anti‐IL‐10 antibodies led to a significant reversal in levels of IL‐13^+^CD4^+^T cells (Fig. 5F), and IL‐9 and IL‐13 protein compared to the sEV‐mDCs alone (Fig. 5G). Moreover, by blocking IL‐10Rα using blocking antibody, the suppressive effects of sEV‐mDC on the levels of IL‐13^+^CD4% but not IL‐9^+^CD4% were significantly reversed (Supporting Information Fig. S6B). Generally, these data demonstrated the inhibitory effects of sEV‐mDCs on the type 2 responses partially by acting on the IL‐10/IL‐10Rα axis, further indicating the potential application of the cell‐free therapy of MSC‐sEV in the treatment of allergic rhinitis.

### sEV‐mDCs promote the expansion of Treg cells

**Figure 6:**
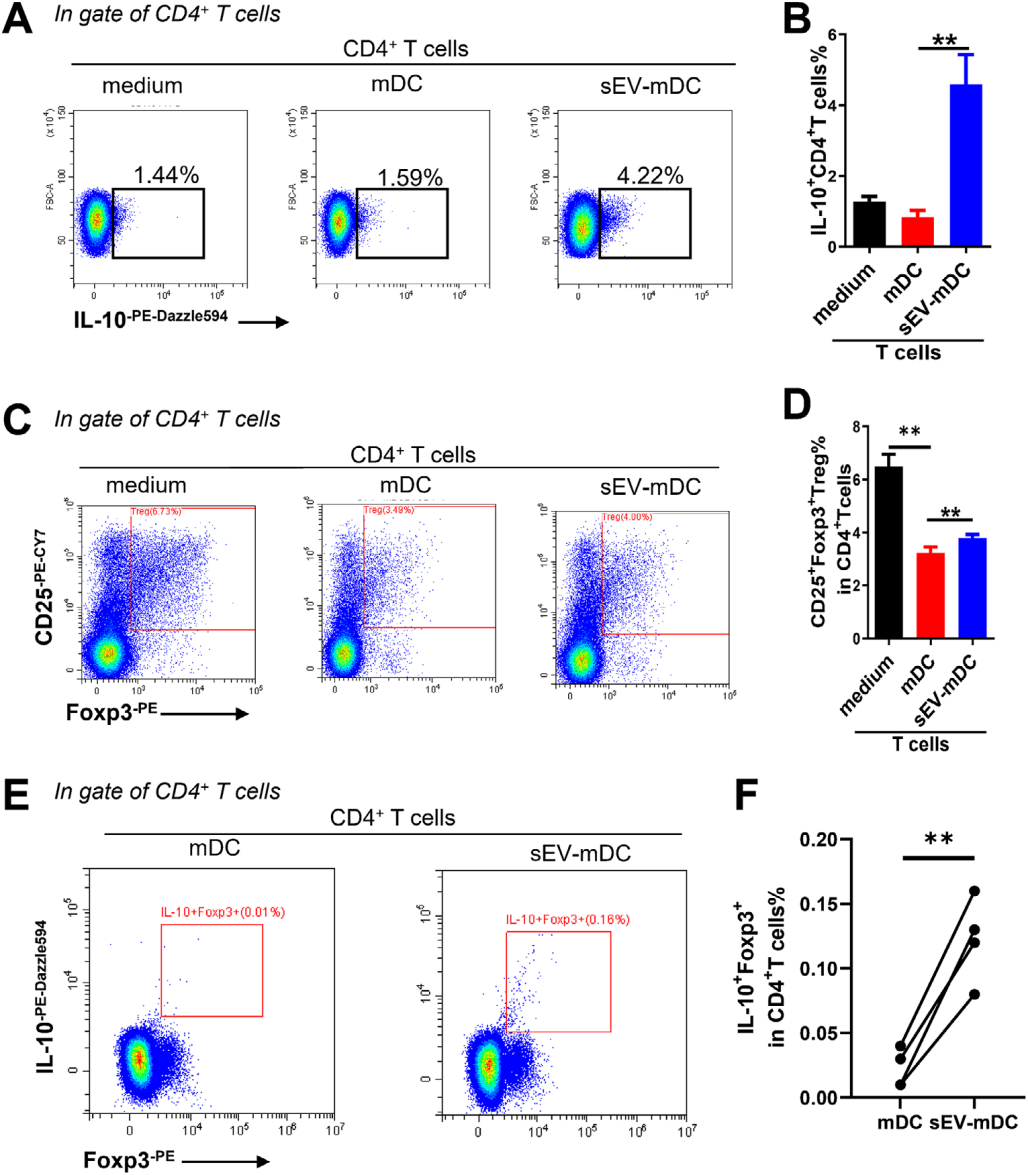
sEV‐mDCs increased the level of Treg cells. Sorted CD4^+^ T cells from buffy coat were co‐cultured with or without mDCs for 3 days. (A and B) The level of IL‐10^+^ T cells in the absence or presence of mDCs treated with or without MSC‐sEV. (C and D) The proportion of CD25^+^Foxp3^+^ Treg cells after the co‐culture. (E and F) The percentages of IL‐10^+^Foxp3 in CD4^+^ T cells after the co‐culture. Data in (A) and (B) are from the same experiments of Fig. 5B. Data are representative of collated data of four donors for T‐cell isolation and four donors for DC generation (C and D). Data in (E) and (F) are from four donors for T‐cell isolation and DC generation. All from buffy coat (mean ± SEM). **p < 0.01, Tukey's multiple comparisons test. mDCs: mature dendritic cells; MSC: mesenchymal stromal cells; sEV: small extracellular vesicles; Treg: regulatory T.

Previous studies have reported Treg deficiency in AR [27], and Treg cells have the ability to decrease Th2 responses [28]. IL‐10 was one of the main functional cytokines of Treg cells to exert their inhibitory effects. Here, we observed a significantly up‐regulated proportion of IL‐10^+^CD4^+^ T cells after CD4^+^ T cells were co‐cultured with sEV‐mDCs (Fig. 6A and B). We further detected the level of Treg cells after co‐cultured with DCs. We found that the level of CD4^+^CD25^+^Foxp3^+^ Treg cells decreased after co‐cultured with mDCs. However, there were higher levels of CD4^+^CD25^+^Foxp3^+^ Treg cells after the administration of sEV‐mDCs compared to mDCs (Fig. 6C and D). Similarly, the level of IL‐10^+^Foxp3^+^CD4^+^T cell was significantly upregulated in response to sEV‐mDCs compared to mDCs (Fig. 6E and F). These data indicate the upregulation of Treg by sEV‐mDCs, which might further contribute to the downregulation of Th2 responses.

## Discussion

In this study, we identified that MSC‐sEV suppressed the differentiation of monocytes into DCs, and MSC‐sEV rendered mDCs with a stronger uptake ability but without affecting their maturation surface markers. Moreover, mDCs treated with MSC‐sEV inhibited the Th2 response through IL‐10, and upregulated the levels of Treg cells. This study enlightened the prospective application of MSC‐sEV in allergic diseases.

MSCs derived from iPSCs have previously been well shown to manifest potent immunoregulatory ability through different mechanisms. However, there are still some limitations of MSCs in clinical application including allogeneic immune rejection, the potential tumorigenicity, and putative profibrogenic potential. Thus, MSC‐sEV have become increasingly promising, in consideration of their features.

Currently, differential ultracentrifugation is most widely used for the isolation of sEV. However, it has been well acknowledged that this method was not applicable for the mass production of MSC‐sEV, which limits the feasible clinical application. As our previous study, anion exchange chromatography, serving as an efficient and scalable method, enabled us to isolate high‐purity MSC‐sEV in future clinical usage [21]. In the foregoing study, we demonstrated the therapeutic effects of MSC‐sEV on the ILC2 function via miR‐146a‐5p in allergic airway inflammation [21]. In addition, EVs derived from BM‐MSCs have been reported to be capable of inducing regulatory dendritic cells in type 1 diabetic patients [29]. Nevertheless, the interaction of the DC‐T cells orchestrated the adaptive immune responses in the pathogenesis of AR. In this regard, it needs to investigate whether MSC‐sEV could attenuate the allergic inflammation through the DC‐T cells pathway.

To verify the effects of MSC‐sEV on the differentiation and maturation DCs, we first generated the sEV‐iDCs and sEV‐mDCs, respectively. According to the expression of DC markers including CD11c, HLA‐DR, CD40, CD80, and CD86, we elucidated that the administration of MSC‐sEV inhibited the differentiation of CD14^+^ monocytes into DCs but without the effects on the surface markers in their maturation, which was consistent with our previous work of the effects of iPSC‐MSCs on DCs [5]. It is worth noting that, despite the inability of MSC‐sEV to affect the maturation of mDCs, it indeed promoted their phagocytic capacity, suggesting that human MSC‐sEV affected the function of mDCs by inducing mDCs to acquire some immunomodulatory properties of regulatory DCs.

mDCs play critical roles in antigen presentation, driving naïve T cells into T helper cells under distinct conditions and promoting the proliferation of T cells in MLC. To figure out the effects of MSC‐sEV on the mDCs to stimulate T cells, we co‐cultured sEV‐mDCs with sorted CD4^+^ T cells. We found that there was no difference in T‐cell proliferation whether co‐cultured with sEV‐mDCs or mDCs. Further, we examined the effects of sEV‐mDCs on Th2 immune responses under the allergic environment. We found a significant decrease in the percentage of IL‐13^+^ T cells and IL‐4^+^ T cells in PBMCs from patients with AR after co‐cultured with sEV‐mDCs. Together, these findings elucidated that MSC‐sEV could attenuate the DC function to inhibit the Th2 enhancing ability under the allergic condition. However, controversial findings have been reported that there was a markedly reduced level of IFN‐γ from healthy controls under the stimulation of sEV from BM‐MSCs, suggesting that sEV‐DCs may skew the balance of Th1 and Th2 effector T cells in favor of the latter [22]. Previous studies have shown that MSCs exert variable immunomodulatory effects on the same types of immune cell depending on the local microenvironment or disease status [30]. The controversial findings may due to different samples (healthy controls or patients) and even different sources of sEV (iPSC‐MSCs or BM‐MSCs). To our best knowledge, it is the first study to investigate the suppression of Th2 immunity exerted by sEV‐mDCs in patients with AR in vitro. We acknowledged that autologous mDCs‐T cells coculture and in vivo study were needed to further elucidate the effects of sEV‐mDC on Th2 response and T‐cell proliferation in patients with AR.

As for the mechanism, we observed a markedly increased level of IL‐10 mRNA in mDCs treated with MSC‐sEV. Moreover, the expression of IL‐12p70 showed a tendency for higher expression in sEV‐mDCs compared to mDCs. It has been intensively reported that DCs exhibited their function through both cell–cell contact and cytokine secretion. IL‐10 was the vital cytokines for the functional regulatory DCs. In the meantime, IL‐12p70 could orchestrate the Th1 immunity elicited by type 1 of DCs. These all supported our findings that MSC‐sEV treatment to mDCs skewed the balance of Th1 and Th2, in favor of the former.

To verify our hypothesis, we further determined the protein level of IL‐10 from the sEV‐mDCs and found an increased level of IL‐10 both in the supernatants and intracellular staining. Overall, these findings uncovered that MSC‐sEV promoted mDCs to produce more IL‐10 and induced them to acquire a regulatory phenotype. Furthermore, we found that IL‐10 could attenuate the percentages of IL‐13^+^ T cells and IL‐9^+^ T cells. Next, to confirm the effects of IL‐10 in the functional sEV‐mDCs, we added the anti‐IL‐10 and IL‐10Rα blocking antibodies to the co‐cultures. As expected, we identified that the blockade of the IL‐10 and IL‐10Rα reversed the suppressive effects of sEV‐mDCs on Th2 response by reducing the production of IL‐9 and IL‐13, and the levels of IL‐13^+^CD4^+^ T cells.

Treg cells were reported to control Th2‐mediated allergic inflammation. We then found that the proportion of IL‐10^+^CD4^+^T cells was 10‐fold upregulated after co‐cultured with sEV‐mDCs. As one of the main sources of IL‐10 producing T cells was Treg cells, we further examined the level of CD4^+^CD25^+^Foxp3^+^ Treg cells. As expected, the percentage of Treg cells was significantly higher in the co‐cultures of T cells and sEV‐mDCs than with mDCs. It suggests that sEV‐mDCs may exert their immunomodulatory function on Th2 cells by the accumulation of Treg cells.

Taken together, our study gives the evidence that sEV derived from human iPSC‐MSCs suppressed the Th2 priming ability of mDCs via IL‐10 in patients with AR in vitro. In this study, we give a new understanding of the cell‐free therapy of MSC‐sEV in the pathogenesis of AR.

## Materials and methods

### Subjects

A total of five patients with AR were enrolled (Table 1). The inclusion criteria for patients with AR were established according to the criteria of the Initiative on Allergic Rhinitis and its Impact on Asthma (ARIA) based on the nasal and/or ocular hypersensitivity symptoms and specific immunoglobulin E (sIgE) tests [31]. The subjects were excluded if pregnant, current smokers, or ex‐smokers with more than 10 pack‐years. None of the subjects has used oral or nasal corticosteroid or other treatments (e.g., H1‐antihistamine or immunotherapy) for 6 weeks before the study. The study was approved by the Ethics Committee of The First Affiliated Hospital, Sun Yat‐sen University, China. Human blood buffy coats (*n* = 47) from “anonymous donors” were from Guangzhou Blood Center; exemption of written informed consent was approved.

**Table 1:** The characteristics of the subjects’ demographics

Characteristic	Allergic rhinitis
No. of patients	5
Age (years)	25.2 ± 3.15
Gender, female/male	3/2
SPT, positive/subjects tested	5 (100%)
tIgE concentration (IU/mL)	115.3 ± 22.51
sIgE concentration (IU/mL)	
Der p/Der f	23.83 ± 5.11
House dust	0.75 ± 0.36
Trees	<0.35
Artemisia/ragweed	<0.35
Molds	<0.35
Hair or dander of pets	<0.35
VAS	24 ± 1.73
TNSS	6.4 ± 0.68

SPT: skin prick tests. sIgE: specific immunoglobulin E. tIgE: total immunoglobulin E. TNSS: total nasal symptom score. VAS: visual analogue scale.

### MSC‐sEV preparation

The MSC‐sEV‐enriched medium in this study was prepared as described in our previous study [21]. iPSC‐MSCs were plated at a density of 1 × 10^6^ cells/dish in 150 mm diameter cell culture dishes with complete culture medium. After the cells reached 70%–80% confluency (about 1 × 10^8^ cells in total), the complete culture medium was replaced with chemically defined and protein‐free medium after three washes with phosphate‐buffered saline (PBS). Chemically defined and protein‐free medium was discarded and then refreshed after 6 h of incubation. After another 42‐h incubation, the sEV‐enriched supernatants were harvested and centrifuged immediately at 2000 *g* for 20 min at 4°C to exclude detached cells and debris.

Isolation of MSC‐sEV was conducted using anion exchange chromatography as our previous report [21]. An Econo‐Pac column (Bio‐Rad Laboratories, CD63 Hercules, CA, USA) was packed with anion exchange resin (GE Health Care Life Science, Pittsburgh, PA, USA) and equilibrated with equilibration buffer. Then the column was loaded with MSC supernatants, washed with wash buffer to remove the proteomic impurities, and eluted continuously with elution buffer for eight times. The fractions with peak concentration of MSC‐sEV were pooled for dialysis overnight at 4°C. Then, we further concentrated the MSC‐sEV using Pierce™ Protein Concentrator (Thermo Fisher Scientific, Rockford, IL, USA), which were used for further analyses and studies. Collected MSC‐sEV were characterized based on their morphology, particle size, and surface protein expression. The final protein and particle concentrations of MSC‐sEV preparations were quantified by Bradford protein assay and nanoparticle tracking analysis (NanoSight NS300; Malvern, UK).

### Transmission electron microscopy for MSC‐sEV

The MSC‐sEV preparations were fixed with 2% glutaraldehyde (Sigma, Saint Louis, MO, USA) for 30 min. The exact procedures could be referred in our previous paper [21, 32]. Micrographs of MSC‐sEV were obtained with a transmission electron microscopy (H7650; HITACHI, Tokyo, Japan) at Guangdong Institute of Microbiology.

### Western blot analysis

The protein concentration of MSC‐sEV and MSC lysates were quantified using the Pierce™ BCA protein assay (Thermo Fisher Scientific, Rockford, IL, USA). Western blot was performed as described previously [21, 32]. CD9 (1:2000), CD63 (1:2000), CD81 (1:2000), Alix (1:5000), TSG101 (1:1000), and Calnexin (1:1000) were used as primary antibodies, all from Abcam, Cambridge, UK. All secondary antibodies were purchased from Jackon ImmunoResearch, West Grove, PA, USA. Finally, the figures were obtained using an imaging analysis system (ImageQuant LAS 4000, GE Health Care Life Science, Uppsala, Sweden).

### Generation of sEV‐immature DCs and sEV‐mature DCs in vitro

Like our previous study, CD14^+^ monocytes from PBMCs in the buffy coat “anonymous donors” provided by Guangzhou Blood Center were isolated using the MACS CD14 MicroBeads (Miltenyi Biotec, Bergisch Gladbach, Germany) [5, 33]. And then the CD14^+^ monocytes were stimulated with recombinant human granulocyte‐macrophage colony‐stimulating factor (GM‐CSF; 50 ng/mL; PeproTech Inc., Rocky Hill, NJ, USA) and IL‐4 (10 ng/mL; R&D Systems, Minneapolis, MN, USA) with or without the administration of MSC‐sEV (5 μg/mL, 10 μg/mL or 20 μg/mL) for 5 days to get iDCs or sEV‐iDCs. On the other hand, the iDCs on day 5 were then stimulated with LPS (100 ng/mL; Sigma‐Aldrich, St Louis, MO, USA) and MSC‐sEV (5–20 μg/mL) for an additional 2 days to be sEV‐mDCs. And the iDCs without the treatment of LPS were harvested on day 7 as iDC‐D7. The DCs were used in the examination of the surface markers by flow cytometry or co‐cultures.

### Phagocytosis assay

To compare the phagocytic ability of DCs, iDC‐D7, mDCs, and sEV‐mDCs were incubated with FITC‐conjugated dextran (mol wt 4000, 100 μg/mL; Sigma‐Aldrich) for 1 h at 37°C, or at 4°C as a negative control. The cells were then washed twice and re‐suspended in ice‐cold PBS for immediate analysis by flow cytometry. The results were shown as mean ∆MFI_(37‐4°C)_ ± SEM.

### Co‐culture experiments of sEV‐mDCs with PBMCs or T cells

Human PBMCs from patients with AR or buffy coat from “anonymous donors” were isolated by density centrifugation with Ficoll‐Paque Plus (MP Biomedicals, Santa Ana, CA, USA). CD4^+^ T cells were sorted from PBMCs of buffy coat of human volunteers using the CD4 Microbeads (Miltenyi Biotec, Bergisch Gladbach, Germany). sEV‐mDCs were harvested and washed twice, and then were co‐cultured with allogeneic PBMCs isolated from patients with AR or freshly sorted CD4^+^ T cells (1:10) for 5 days in RPMI 1640 (Hyclone, Pittsburgh, PA, USA) supplemented with 10% FBS and IL‐2 (100 U/mL). Cell counting was performed manually using a hemocytometer and the cell viability was over 95%. In some experiments, CD4^+^ T cells were treated with the rhIL‐10 (10 ng/mL; PeproTech Inc.) together with mDC, or incubated with anti‐IL‐10 monoclonal antibody (75 ng/mL) or IL‐10Rα blocking antibody (5 μg/mL; both from R&D systems, Minneapolis, MN, USA) for 30 min before the co‐culture. The PBMCs or the T cells were collected for the analysis of intracellular Th2 cytokines using flow cytometry, and the supernatants were used for the evaluation of IL‐13, IL‐9, IL‐10, or IFN‐γ. T cells were also evaluated for IL‐10^+^Foxp3^+^ T cells or Treg cells.

### T‐cell proliferation

For the analysis of cell proliferation, freshly sorted CD4^+^ T cells stained with carboxyfluorescein diacetate succinimidyl diester (Invitrogen/Molecular Probes, Eugene, Ore) were cocultured with mDCs or sEV‐mDCs for 5 days, and the proliferation rate was evaluated on a CytoFLEX S flow cytometer. The data were analyzed with CytExpert (Beckman Coulter).

### Flow cytometry

DCs were stained with the following specific mAbs: FITC‐conjugated lineage cocktail: anti‐CD2 (RPA‐2.10), anti‐CD3 (OKT3), anti‐CD14 (61D3), anti‐CD16 (CB16), anti‐CD19 (HIB19), anti‐CD56 (TULY56), anti‐CD235a (HIR2), eFluor 450‐conjugated anti‐CD45 (HI30), PE‐conjugated anti‐CD11c (BU15), APC‐conjugated anti‐CD40 (5C3), FITC‐conjugated anti‐CD80 (2D10.4), PE‐Cyanine7‐conjugated anti‐CD86 (IT2.2, all from eBioscience, San Diego, CA, USA), and APC‐H7‐conjugated anti‐HLA‐DR (G46‐6; BD Pharmingen, Bergen, NJ, USA). T cells and Treg cells were assessed using the antibodies of Fixable Viability Dye eFluor™ 506, PerCP‐Cyanine5.5‐conjugated anti‐CD4 (RPA‐T4), PE‐Cyanine7‐conjugated anti‐CD25 (BC96; all from eBioscience). Detailed gating strategies are shown in Supporting Information Fig. S2.

For intracellular cytokine detection, PBMCs or T cells were stimulated with the cell stimulation cocktail consisting of phorbol 12‐myristate 13‐acetate (50 ng/mL), ionomycin (1000 ng/mL; both from Sigma), and monensin (1:1000; eBioscience) for 5 h prior the staining. Thereafter, the cells were fixed, permeabilized with intracellular fixation and permeabilization buffer set (eBioscience), and stained for intracellular IL‐13 (V450‐conjugated anti‐IL‐13; JES10‐5A2), IL‐9 (Alexa Fluor 647‐conjugated anti‐IL‐9; MH9A3; both from BD Pharmingen, Bergen, NJ, USA), and IL‐4 (APC‐conjugated anti‐4; 8D4‐8; eBioscience). And the intracellular IL‐10 in DCs or T cells was determined using the antibody of PE‐Dazzle594‐conjugated anti‐IL‐10 (JES3‐9D7, BioLegend, San Diego, CA, USA).

T cells were processed with the FoxP3/Transcription Factor Staining Buffer Set (eBioscience) before staining with the antibody of PE‐conjugated anti‐Foxp3 (PCH101; eBioscience) for the evaluation of Treg cells. Flow cytometry were performed and presented, adhered to the guidelines [34].

### ELISA

The cell supernatants were analyzed by using IL‐13, IL‐9, and IL‐10 ELISA kits (Invitrogen, Waltham, MA, USA), and IFN‐γ ELISA kits (RayBiotech, Guangzhou, Guangdong, China), according to the manufacturer's instruction.

### RT‐qPCR

Total RNA was isolated from mDCs with RNAiso Plus reagent and the 5X PrimeScript™ RT Master Mix kit (all from TaKaRa, Shiga, Kusatsu, Japan) was used for converting 1 μg of total RNA to the first‐strand cDNA following the manufacturer's instructions. The quantitative PCR of IL‐10 (sense primers, 5′‐GACTTTAAGGGTTACCTGGGTTG‐3′, and reverse primer, 5′‐TCACATGCGCCTTGATGTCTG‐3′) were performed using the FastStart Universal SYBR Green Master kit (Roche, Mannheim, Germany). β‐Actin (sense primers, 5′‐AGAGCTACGAGCTGCCTGAC‐3′, and reverse primer, 5′‐ AGCACTGTGTTGGCGTACAG‐3′) was used as an endogenous reference. The PCR was performed as 10 min initial denaturation at 95°C, 40 cycles consisted of 10 s at 95°C and 30 s at 60°C carried out on the CFX96™ Real‐Time PCR cycler (Bio‐Rad, Hercules, CA, USA). The expressions of the target genes were expressed as fold increase relative to the expression of β‐actin. The mean value of the replicates for each sample was calculated and expressed as cycle threshold (CT). The amount of gene expression was then calculated as the difference (ΔCT) between the *Ct* value of the target genes and the *Ct* value of β‐actin. Fold changes in target genes mRNA were determined as 2^−ΔCT^.

### Statistical analyses

Differences between two groups were analyzed by the paired *t*‐test for the data with normal distribution, and the Wilcoxon matched‐pairs signed‐rank test was performed to compare the data with abnormal distribution. Three or more groups were compared using one‐way analysis of variance (ANOVA) with Tukey's multiple comparisons test. Kolmogorov–Smirnov test was used to test the normality of all the data. *p* < 0.05 was considered statistically significant. Analyses were performed using GraphPad Prism 8.0 software (GraphPad Software, La Jolla, CA, USA).

### Ethics approval

The study was approved by the Ethics Committee of The First Affiliated Hospital, Sun Yat‐sen University, China (No. [2017]138). Signed informed consents and required documentation were obtained from each patient and respective guardian prior to the study. Human blood buffy coats from “anonymous donors” were from Guangzhou Blood Center; exemption of written informed consent was approved.

## Conflict of interest

The authors declare no commercial or financial conflict of interest.

## Author contributions

YQP performed the experiments and helped in the collection and assembly of data, initial manuscript writing, and primary data analysis. ZCW and ZBX contributed to perform the experiments. SBF prepared the MSC‐sEV and carried out electron microscopy analysis of MSC‐sEV. DHC and DC helped to enroll the patients with AR. HYZ, XQL, and BXH helped in collection and/or assembly of data. QLF contributed to concept and design of the study, and helped in manuscript writing and final approve the manuscript. All authors read and approved the manuscript.

### Peer review

The peer review history for this article is available at https://publons.com/publon/10.1002/eji.202149497

**Abbreviations**
AR: allergic rhinitis
ILC2: group 2 innate lymphoid cell
iPSCs: induced pluripotent stem cells
MSCs: mesenchymal stromal cells
MSC‐sEV: MSC‐derived small extracellular vesicles
sEV‐iDCs: sEV‐immature DCs
sEV‐mDCs: sEV‐mature DCs

## Supporting information

Supporting Information

## Data Availability

The data that support the findings of this study are available from the corresponding author upon reasonable request. The data are not publicly available due to privacy or ethical restrictions.
