# Supplementary material for: Mesenchymal stromal cells‐derived small extracellular vesicles modulate DC function to suppress Th2 responses via IL‐10 in patients with allergic rhinitis
Source: Eur J Immunol. 2022 Apr 24;52(7):1129–40. doi: 10.1002/eji.202149497 (PMC9545324; doi:10.1002/eji.202149497)
Supplement: Supplementary file 1 — Supporting Information [file EJI-52-1129-s001.pdf]

Fig. S1

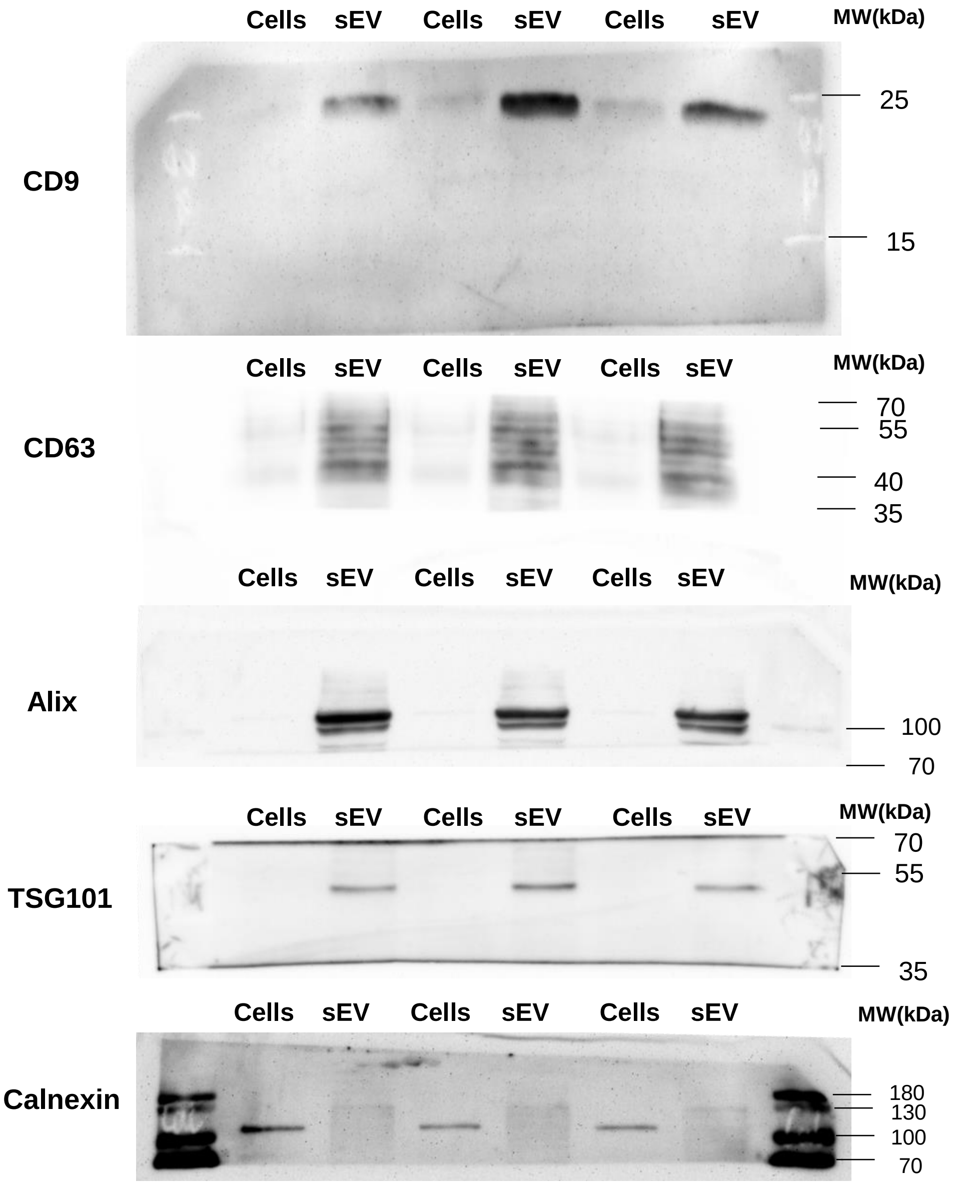

Figure S1. The definite expressions of EV markers by Western Blot. CD9, CD63, Alix and TSG101 were positive and Calnexin was negative in the sEV derived from MSCs as determined by Western Blot. The raw data were shown above.

Fig. S2

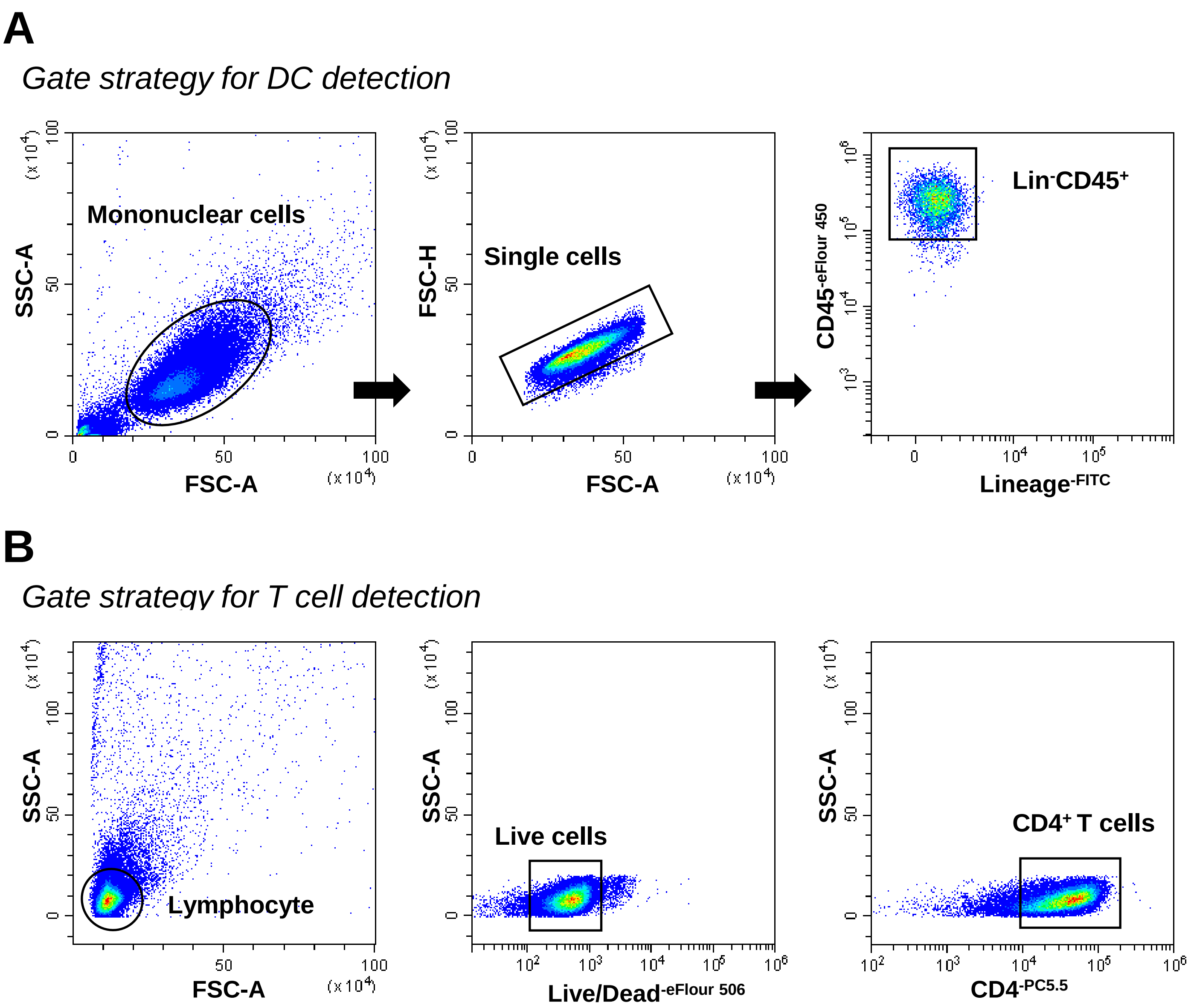

Figure S2. The Gating strategy for DC (A) and T cell (B) detection. DCs were determined in the gate of Lin⁻CD45⁺ cells. T cells were determined in the gate of CD4⁺ cells.

Fig. S3

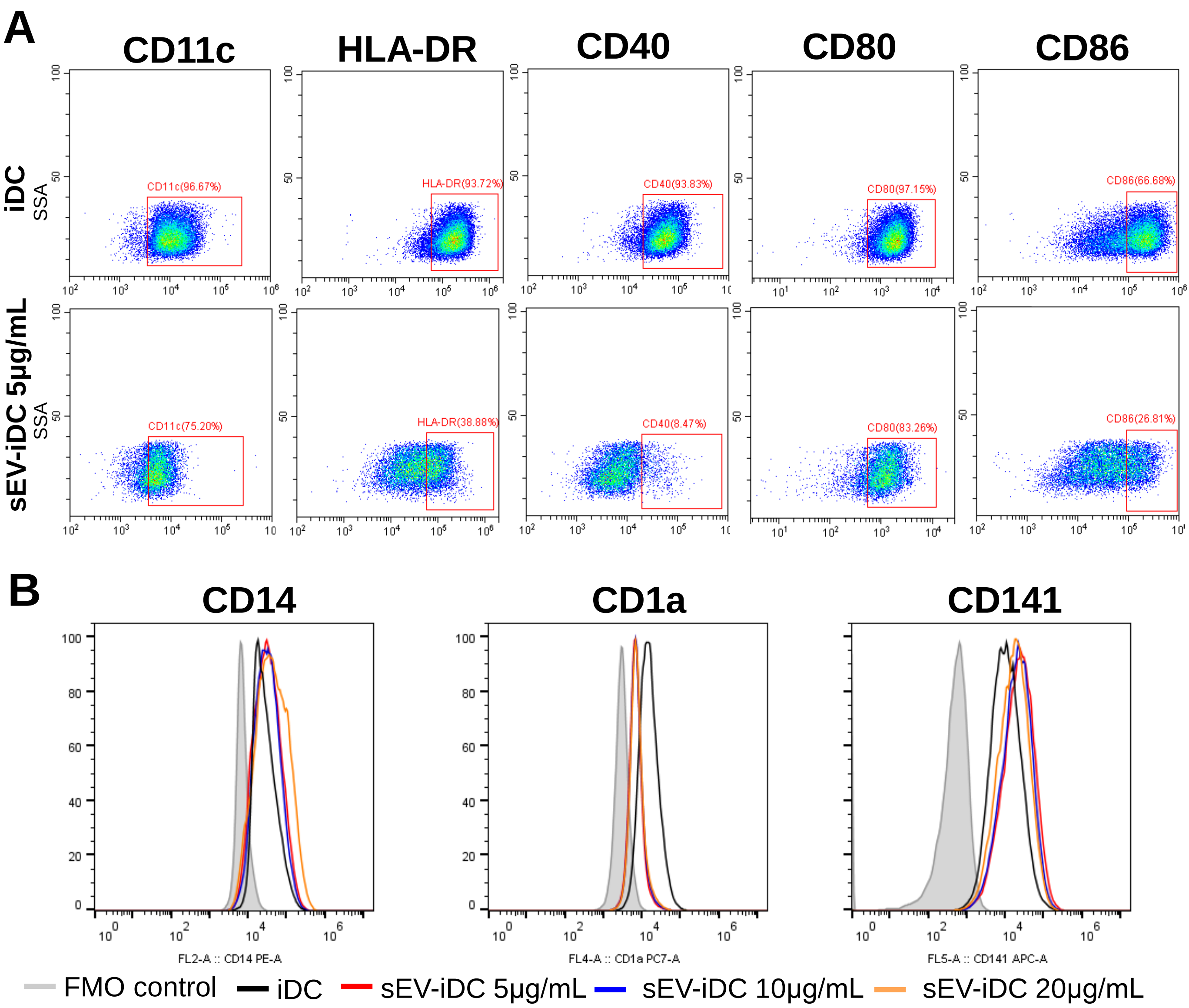

Figure S3. The representative pictures of MSC-sEV on the differentiation of DCs. $CD14^+$ monocytes were cultured in the presence or absence of MSC-sEV (5, 10 or 20 μg/mL) for 5 days. **A,** Dot plots of markers (CD11c, HLA-DR, CD40, CD80, and CD86) were determined by flow cytometry. **B,** The expression of CD14, CD1a, and CD141 was determined by flow cytometry. DCs: dendritic cells, FMO: fluorescence minus one, MSC: mesenchymal stromal cells, sEV: small extracellular vesicles.

Fig. S4

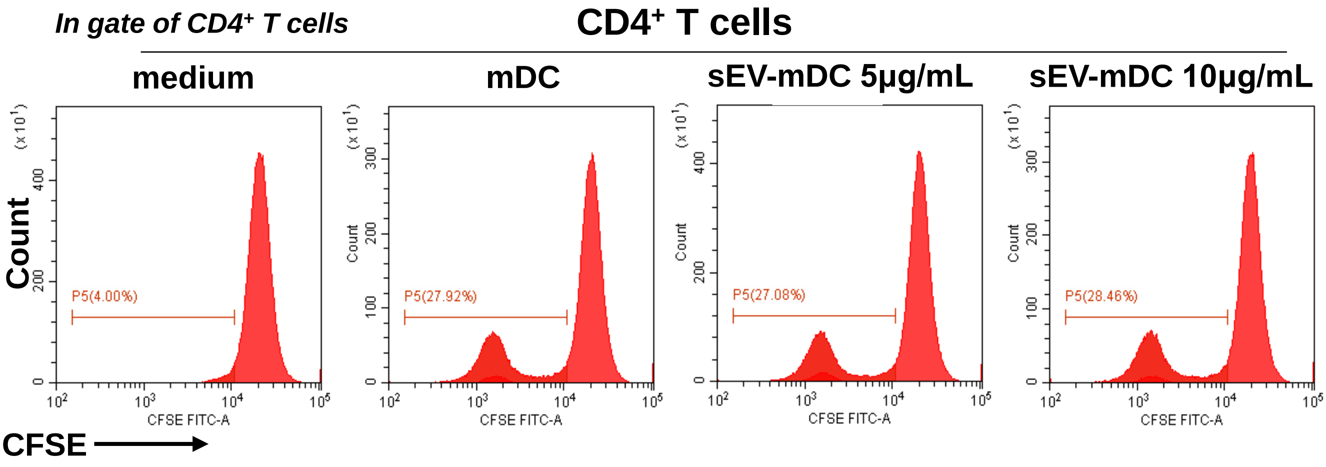

Figure S4. MSC-sEV did not affect the promoting ability of mDCs on T cell proliferation. CFSE-labeled $CD4^+$ T cells were co-cultured with or without mDCs and sEV-mDCs for 5 days. The proliferation rate of T cells was determined by flow cytometry. CFSE: carboxyfluorescein succinimidyl ester, mDCs: mature dendritic cells; MSC: mesenchymal stromal cells; sEV: small extracellular vesicles.

Fig. S5

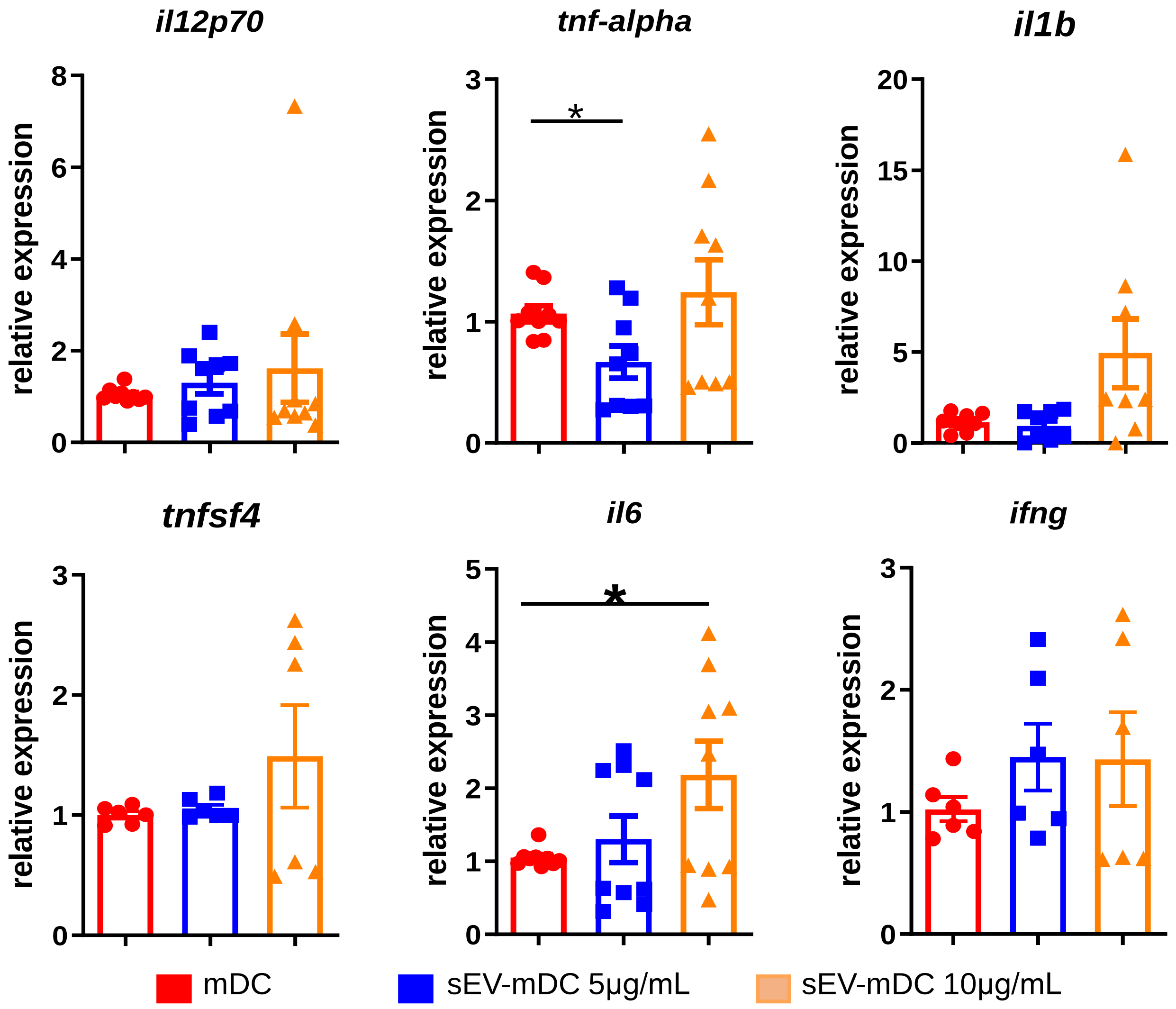

Figure S5. The levels of IL-12p70, TNF-α, IL-1β, OX-40L (encoded by *tnfsf4*), IL-6 and IFN-γ mRNA in mDCs treated with or without sEV. Data are shown as mean ± SEM (n = 6 - 9). *$P < 0.05$. mDCs: mature dendritic cells; sEV: small extracellular vesicles.

Fig. S6

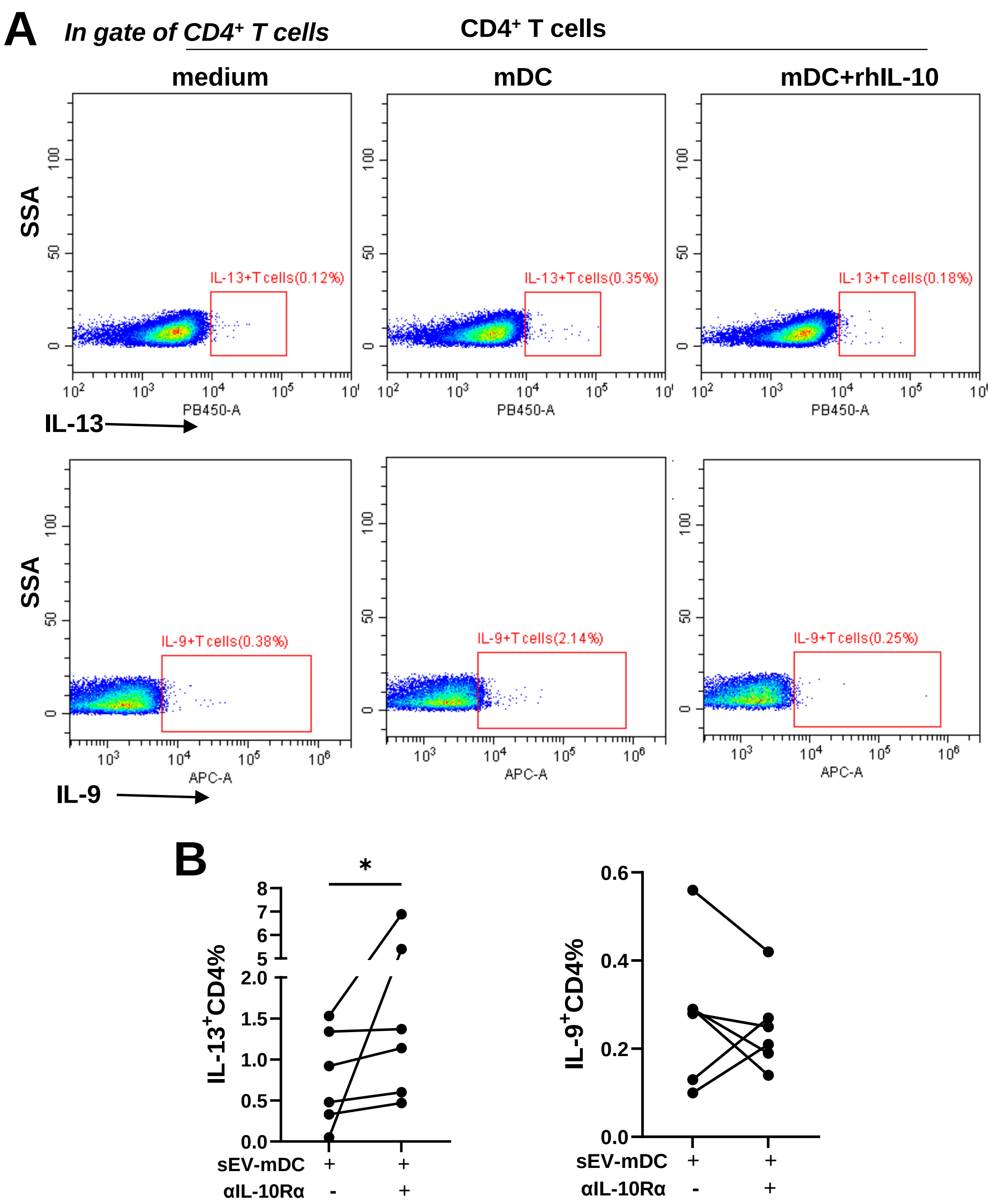

Figure S6. IL-10 was responsible for the immunomodulatory effects of sEV-mDCs on T cells. A, The dot plots of intracellular IL-9 and IL-13 in T cells in the addition of rhIL-10 (10 ng/mL) antibody to the co-cultures (n = 6). **B,** The bar graphs of intracellular IL-9 and IL-13 in T cells with the treatment of anti-IL-10Rα blocking antibody (5 μg/mL) to the co-cultures (n = 6). Data are representative of collated data of six donors for T cell isolation and three donors for DC generation, all from buffy coat (mean ± SEM). **P* < 0.05, paired t-test. mDCs: mature dendritic cells; MSC: mesenchymal stromal cells; sEV: small extracellular vesicles.
